# Twenty years of sex and gender science in health research: a policy statement from the Organization for the Study of Sex Differences

**DOI:** 10.1186/s13293-026-00894-w

**Published:** 2026-05-12

**Authors:** Sofia B. Ahmed, Jill B. Becker, Katelyn A. Bruno, Sandra M. Dumanski, Liisa A. M. Galea, Marija Kundakovic, Armin Raznahan, Natalie C. Tronson, Rebecca L. Cunningham

**Affiliations:** 1 Organization for the Study of Sex Differences, Executive Committee, Bandera, TX USA; 2 Department of Medicine, Faculty of Medicine and Dentistry, University of Alberta, Edmonton, Canada; 3 Women and Children’s Health Research Institute, University of Alberta, Edmonton, Canada; 4 Department of Psychology, Michigan Neuroscience Institute, University of Michigan, Ann Arbor, MI USA; 5 Division of Cardiovascular Medicine, Department of Medicine, University of Florida, Gainesville, FL USA; 6 Department of Medicine, Cumming School of Medicine, University of Calgary, Calgary, Canada; 7 Libin Cardiovascular Institute, University of Calgary, Calgary, Canada; 8 O’Brien Institute for Public Health, University of Calgary, Calgary, Canada; 9 Treliving Family Chair in Women’s Mental Health, Centre for Addiction and Mental Health, Toronto, ON Canada; 10 Department of Psychiatry, University of Toronto, Toronto, ON Canada; 11 Department of Biological Sciences, Fordham University, Bronx, NY USA; 12 Section On Developmental Neurogenomics, NIMH Intramural Research Program, Bethesda, MD USA; 13 Department of Pharmaceutical Sciences, College of Pharmacy, University of North Texas Health Science Center, Fort Worth, TX USA

## Abstract

Sex and gender are fundamental determinants of health, disease risk, and treatment responses, yet they remain inconsistently and inadequately integrated into biomedical research. Despite major funding and regulatory policies over the past two decades, sex and gender continue to be treated primarily as descriptive variables rather than drivers of discovery. As the Organization for the Study of Sex Differences (OSSD) marks twenty years of leadership in this field, this policy statement articulates OSSD’s position on best practices for the integration of sex and gender in health research. Drawing on empirical evidence, landmark policies, and persistent gaps in implementation, this statement provides clear guidance for researchers, funders, and journals. OSSD explicitly recommends prespecified sex- and gender-responsive questions, appropriate study design and statistical power, transparent analysis and reporting, and accountability mechanisms to translate into practice. Adoption of these standards is essential to improving scientific rigor, reproducibility, and clinical relevance.

## Policy rationale and scope

As the Organization for the Study of Sex Differences (OSSD) commemorates 20 years of leadership and impact in the field of sex and gender science, this moment provides an opportunity to reflect on how far the field has come and how much work remains. Over the past two decades, a growing body of evidence has demonstrated that sex and gender have profound effects on biology, disease pathophysiology, health outcomes, health behaviors, and lived experiences. These considerations are no longer optional or supplementary, but are foundational to scientific rigor, health equity, and the precision and impact of biomedical research.

OSSD is committed to facilitating sex and gender research and supporting the translation of this knowledge to improve health and health care. Accordingly, we provide clear, evidence-informed guidance for integrating sex and gender considerations across the full research lifecycle, including study design, methodology, analysis, reporting, and knowledge mobilization. Specifically, this statement addresses definitions of sex and gender, the evolution and growing evidence base of the field, the global policy and regulatory landscape, persistent implementation gaps, the scientific and clinical consequences of neglecting sex and gender, and concludes with policy recommendations and OSSD’s call to action on sex- and gender-responsive research. Collectively, this document aims to promote rigorous, equitable sex- and gender-responsive research that informs practice, policy, and scientific advancement.

## Definitions of sex and gender

Although definitions of sex and gender have evolved and remain dynamic, there is increasing consensus regarding how these concepts are defined, distinguished, and applied in research and health contexts. OSSD recognizes ongoing evolution in terminology and adopts the following working definitions.

- **Sex** refers to biological attributes of living organisms, including but not limited to chromosomal complement, gene expression, endogenous hormone profiles, reproductive and sexual anatomy, and physiological characteristics. These attributes influence development, physiology, and disease risk.
- **Gender** refers to the multidimensional sociocultural roles, behaviors, identities, expressions, and institutional structures that shape how individuals and groups experience and interact with society. Gender influences health through mechanisms such as social roles, norms, access to resources, discrimination, and lived experiences.

While conceptually distinct, sex and gender are deeply entangled and often not easily separable in real-world contexts [1]. This entanglement has important implications for measurement, categorization, causal inference, and scientific transparency. In addition, sex and gender interact with other social and structural factors (e.g., race, Indigeneity, socioeconomic position, disability, and geography [2], underscoring the importance of intersectional approaches to research [3]. Incorporating these perspectives improves the relevance, precision, and equity of scientific findings for diverse communities [4]. We acknowledge that sex and gender terms are often inappropriately conflated. To avoid assumptions on our part, the terminology used throughout this position statement reflects that of the cited studies.

## Historical context: sex and gender research prior to OSSD

**Fig. 1:**
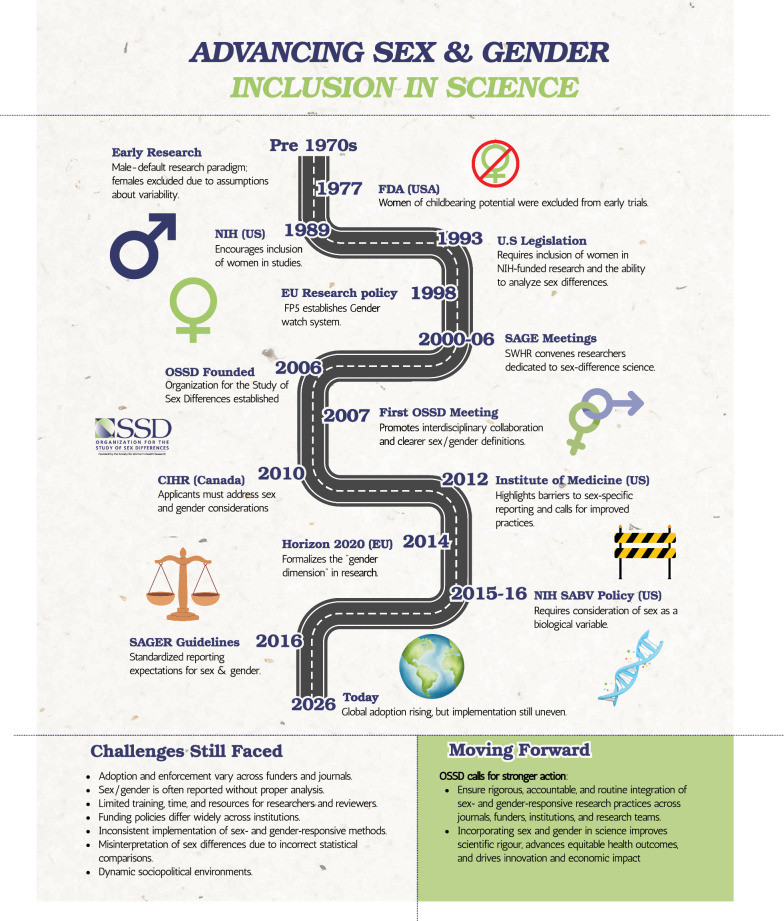
Advancing Sex And Gender Inclusion in Science

Historically, sex and gender were largely neglected in biomedical research [5]. To the best of our knowledge, we have not identified formal guidance addressing sex- or gender-related inclusion in health research issued prior to 1977 (Fig. 1). In 1977, the U.S. Food and Drug Administration (FDA) recommended excluding women of childbearing potential from Phase I and early Phase II trials, framing the policy as a protective response to fetal-risk events such as those associated with the medication thalidomide [6]; this guidance contributed to women being widely excluded from early drug studies until policy reversals in the early 1990s [7]. Although the U.S. National Institutes of Health (NIH) encouraged inclusion of women in studies beginning in 1989, inclusion only became legally required for NIH-funded clinical research under the NIH Revitalization Act of 1993 (Public Law 103–43 titled *Women and Minorities as Subjects in Clinical Research*) [8], which also required trials to be designed to allow a valid analysis of whether effects differed by sex (and by minority group status). Subsequent oversight found inconsistent implementation of inclusion requirements and shortcomings in carrying out the analysis expectations [9, 10]. In 1998, the European Union (EU) Research Policy Fifth Framework Programme (FP5) established the *Gender watch system* aimed at “collecting sex-disaggregated data, encouraging gender research within the framework programmes and conducting gender impact assessment studies on FP5” [11].

Reflecting concerns highlighted in the 2001 National Academies Report *Does Sex Matter?* [5], preclinical research prioritized male organisms as the default experimental model with female organisms commonly underrepresented, often justified by concerns that ovarian/estrous cycling would add variability or complexity [12]. As a result, this practice resulted in a male-derived preclinical evidence base, with knowledge about sex differences accumulated unevenly across biomedical subfields rather than being consistently integrated into generalizable models [5].

In response to these gaps, the Society for Women’s Health Research (SWHR) convened interdisciplinary “Sex and Gene Expression” (SAGE) meetings from 2000–2006. After SWHR concluded its sponsorship of the SAGE meetings, investigators dedicated to advancing sex-differences research across cell-based studies, animal models, and humans founded the Organization for the Study of Sex Differences (OSSD) in 2006 with support from SWHR [13]. The first annual OSSD meeting was held in 2007 in Washington, D.C. with a mission “to enhance knowledge of sex/gender differences by facilitating interdisciplinary communication and collaboration among scientists and clinicians of diverse backgrounds” [14].

The founding of OSSD also reflected broader conceptual and methodological challenges at the time. In the biomedical literature, “sex” and “gender” were frequently used inconsistently or interchangeably, reinforcing the need for clearer definitions and more rigorous measurement [15]. Because validated approaches to assess gender were relatively underdeveloped and unevenly applied in many biomedical contexts, early sex/gender differences research often prioritized sex as the more consistently operationalized variable. At the same time, preclinical studies disproportionately used male cells and animals or failed to report sex, constraining progress on understanding sex differences [16].

## Policy and regulatory landscape

Over the past two decades, major funders around the world have increasingly embedded sex- and gender-based considerations into research expectations. In the European Commission’s (EC) Sixth Framework Programme (2002–2006), research applicants were instructed to address “whether, and in what sense, sex and gender are relevant in the objectives and the methodology of projects”, formally embedding consideration of sex and gender within EU research design and evaluation processes [17]. This emphasis was further formalized under the EC Horizon 2020 (2014–2020) framework, which integrated the “gender dimension” (i.e., sex/gender analysis where relevant) into proposal guidance, evaluation, and monitoring [18]. Under Horizon Europe (2021–2027), formalization strengthened further, where integrating a gender dimension in research and innovations content became a “requirement by default” and is evaluated under the Excellence criterion, unless the topic explicitly specifies otherwise [19]. In 2010, the Canadian Institutes of Health Research (CIHR) required grant applicants to explicitly state whether sex (biological) and/or gender (socio-cultural) considerations were incorporated in proposed research [20], and this was incorporated into peer review criteria in 2018 [21]. In the United States, the NIH articulated a cross-cutting expectation to consider sex as a biological variable (SABV) in study design, analysis, and reporting for vertebrate animal and human studies in a 2015 policy notice, implemented for application due dates in 2016 [22]. The NIH has included the SABV policy within its “rigor and reproducibility” framework, [23] emphasizing that the failure to account for sex can undermine rigor, transparency, and generalizability of research findings, and that single-sex studies require strong scientific justification. At the time of this writing, and noting that this is not an exhaustive review, similar requirements and expectations are also evident across multiple regions worldwide (Table 1).

**Table 1:** Selected International Research Funder Requirements and Expectations for the Consideration of Sex and Gender

Region/Country	Funder / Body	Requirement or Expectation
Australia	National Health and Medical Research Council (NHMRC)	NHMRC and Medical Research Future Fund grant opportunities include a requirement to account for sex, gender, variations of sex characteristics, and sexual orientation in application and assessment processes [24]
Colombia	Ministerio de Ciencia, Tecnología e Innovación	Research protocols must incorporate sex and gender differences, including diverse sexual orientations and gender identities, and consider biological and sociocultural factors that generate health inequities [25]
Japan	Japan Science and Technology Agency	Called for “integration of sex and gender into research and innovation” as a science, technology, and innovation policy direction [26]
Netherlands	ZonMw	Provides tools and guidance to support integration of sex and gender in grant applications and assessments (e.g., FAQs, e-book, reviewer checklist) and promotes integration of sex and gender issues in research and innovation [27]
South Korea	Framework Act on Science and Technology	Amendments introduced explicit consideration of sex and gender in science and technology policy as part of legal and institutional efforts supporting Gendered Innovations research [28]
Taiwan	National Science and Technology Council	Proposal documentation includes explicit application components for sex/gender analysis in clinical trials, including a required response and supporting checklist/reporting attachments [29]
Tanzania	Tanzania Commission for Science and Technology	Guidelines expect proposals to address gender equality and inclusivity in project design, implementation, and reporting [30]
United Kingdom	United Kingdom Research Institute Medical Research Council	Applicants are required “to include both sexes in experimental design for studies involving animals and human/animal tissues and cells, unless there is a strong justification for not doing so,” with supporting guidance [31]
United Kingdom	MESSAGE Project (Medical Science Sex and Gender Equity)	Co-designs and promotes a sex and gender policy framework for UK research funders and regulators, with guidance to support implementation across the research sector [32]
Uruguay	Agencia Nacional de Investigación e Innovación	Applicants must explicitly answer and justify whether sex and/or gender differences are considered across research stages and impacts [33]
Global	Wellcome	Requires applicants to integrate sex and gender throughout the research process where appropriate (design, recruitment, data collection, analysis, reporting), and to provide a strong evidence-based rationale when sex/gender not considered [34]
Global	Gates Foundation	Gender Integration Marker used to assess the level of gender integration within study design [35]

In parallel, reporting and publication standards have evolved to improve transparency. In 2012, the Institute of Medicine released *Sex-Specific Reporting of Scientific Research: A Workshop Summary* [36], highlighting barriers to sex-specific reporting and recommending improved reporting practices, with a particular focus on publications and associated data. In 2016, the Sex and Gender Equity in Research (SAGER) Guidelines [37], developed by the European Association of Science Editors Gender Policy Committee, were launched and promoted as a comprehensive approach to reporting sex- and gender-related information across study design, methods, analysis, and results. These guidelines have been endorsed and supported by multiple publishers, research institutions, editorial and publication ethics societies, and public health organizations including the World Health Organization [38].

Beyond journals and publishers, regulators and health-system governance bodies have reinforced expectations for sex- and gender-aware analysis and reporting, with similar policy directions emerging worldwide. In the United States, the Department of Defense Congressionally Directed Medical Research Programs (CDMRP) has issued a directive establishing requirements to consider SABV across CDMRP-funded research [39], and the U.S. FDA has issued guidance addressing sex differences in medical product evaluation [40], including its longstanding 1993 guidance on the study and evaluation of sex/gender differences in drug development [41] and updated guidance on sex-specific data in medical device clinical studies [42].

Comparable guidance exists in other jurisdictions. In Canada, Health Canada has published guidance on the inclusion of women in clinical trials and the analysis of sex differences [43], complemented by Canada’s Health Portfolio Sex- and Gender-Based Analysis Plus (SGBA +) policy, which emphasizes integrating SGBA + across Health Portfolio activities [44]. In Europe, the European Medicines Agency hosts International Council for Harmonisation guidance addressing gender considerations in clinical trials [45], and in Switzerland, the Swiss Association of Research Ethics Committees has issued recommendations on sex and gender considerations in research involving humans [46].

 Other regional bodies also explicitly promote sex/gender analysis and reporting. PanAmerican Health Organization guidance recommends sex-disaggregated and gender analysis and reporting [47], the African Union has called for sex-disaggregated data to inform gender-responsive health responses [48], and the Association of Southeast Asian Nations has issued guidelines on the use of sex-disaggregated data in crisis and disaster health contexts [49]. The World Health Organization’s “Closing data gaps in gender” initiative further emphasizes collecting, analyzing, and using disaggregated data (including by sex) to identify and respond to gender inequalities in health [50].

## Expansion of the evidence base

**Fig. 2:**
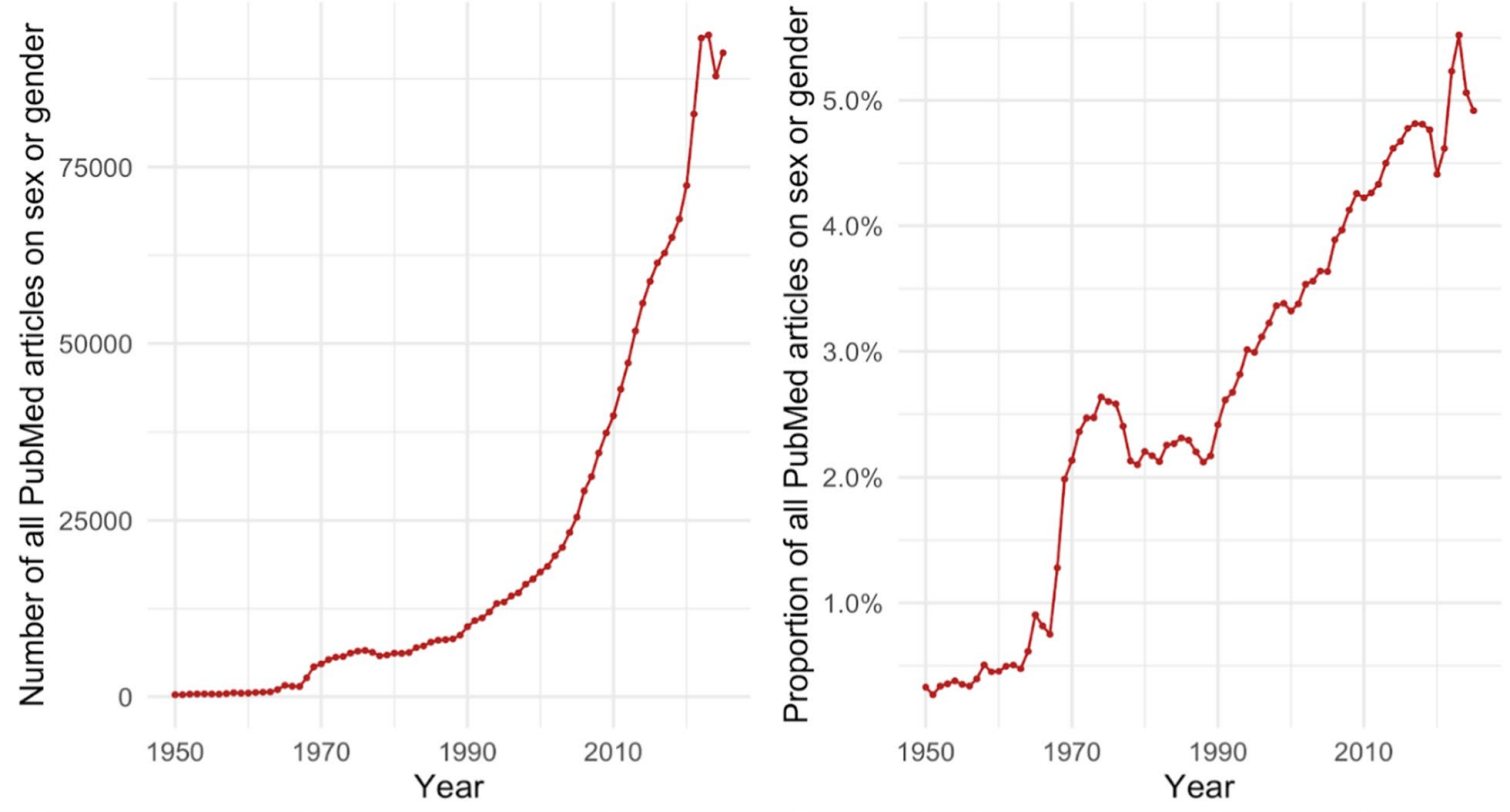
Trends in PubMed-indexed articles that include the terms ‘sex’ or ‘gender’ in bibliographic fields. The left panel shows the total number of articles per year, and the right panel shows the proportion of all PubMed articles per year that include ‘sex’ or ‘gender’ in the title, abstract, MeSH terms, or author-provided keywords. These rates reflect mention or inclusion in indexing and text fields and do not necessarily indicate that an article analyzed sex- or gender-based effects

Over the past 20 years, biomedical research addressing sex and gender has increased substantially, reflecting acceleration of trends that began in the mid-1990s (Figure 2, left panel). This growth persists even when adjusted for overall increases in biomedical publications (Figure 2, right panel), indicating sustained and expanding attention to sex and gender research. This body of evidence underscores the necessity of considering sex in both basic and clinical science while also emphasizing that in human studies, sex-related biology and gendered social factors often interact in complex, inseparable ways. Robust evidence shows that sex and gender differences extend well beyond reproduction and are clinically meaningful across organ systems and the life cycle [51]. The examples below are illustrative and reflect patterns observed across multiple biomedical disciplines.

Within neuroscience, longitudinal neuroimaging and epidemiologic studies demonstrate sex-differentiated trajectories of brain development and maturation [52, 53] along with sex-biased prevalence and clinical phenotypes in neurodevelopment, neuroinflammatory, neurodegenerative and psychiatric disorders, including autism, depression, anxiety disorders, multiple sclerosis, and Alzheimer’s disease [54–56]. In particular, increased risk for depression and anxiety disorders coincides with significant ovarian hormone fluctuations in individuals with ovaries during puberty onset, premenstrually, postpartum, and during the menopausal transition [57]. Cardiovascular disease risk is related to female sex-specific or sex-predominant factors such as adverse pregnancy outcomes, polycystic ovary syndrome, menopause, breast cancer therapy, autoimmune and rheumatic diseases, and depression [58, 59], but also to gender roles such as household-related stress [60]. Cardiovascular diseases may present differently in women compared to men. For example, women may experience angina related to microvascular dysfunction/disease in the absence of obstructive coronary artery disease on coronary angiography [61]. Women experience differences in treatment response and may be at increased risk for adverse treatment effects such as drug-induced QT prolongation and bleeding with antithrombotic therapy, underscoring a need for sex- and gender-aware diagnostic and therapeutic approaches [62]. Emerging epidemiologic evidence further suggests that patterns of cardiovascular disease may also vary across gender identities [63, 64]. Many autoimmune diseases occur more often in women than men, and mechanistic work emphasizes contributions from hormones, chromosomes, and immune regulation [65]. In respiratory disease, asthma is more common in boys before puberty and more common in women in adulthood, underscoring the relevance of both biology and life stage [66]. Extensive evidence documents health disparities among lesbian, gay, and bisexual populations [67–69]. Collectively, these findings highlight the need for more accurate and inclusive methods to measure sex, gender, and sexual orientation across health research and care. In response, more rigorous and inclusive approaches have been developed across research, clinical, and administrative data systems [70, 71] to address the historical exclusion of sexual and gender minority populations in health research and care [72] and the ongoing concern that binary treatment of sex or gender variables in clinical and research systems introduces bias and undermines equity [73, 74].

## Persistent gaps and limitations in implementation

Despite this progress, adoption and enforcement remain variable across funders and journals worldwide. Audits in general medical and global health literature show that sex and gender are inconsistently reported and are often included without corresponding sex-/gender-based analyses [75]. The COVID-19 evidence base, generated at unprecedented speed and scale, demonstrates that these gaps persist even in urgent, high-stakes clinical research [76].

While the SAGER Guidelines provide an editor-facing framework for integrating sex- and gender-related information across study design, methods, analysis, and results [37], implementation remains inconsistent; barriers include limited time, expertise, and capacity across researchers, reviewers, and editorial workflows, alongside perceived costs and effort [77]. Policy coverage also varies among major funders: for example, the Gendered Innovations policy table notes that the U.S. National Science Foundation does not request integration of sex/gender analysis into research design within its proposal/award policies [78].

Even where mandates and guidance exist, implementation of sex- and gender-responsive research practices remain inconsistent. While inclusion of both females and males has increased substantially as reflected in bibliometric analyses showing more frequent sex-inclusive study designs across biological disciplines, only a small proportion of studies use sex or gender as meaningful discovery variables [79–83]. Women’s representation in randomized controlled trials (RCTs) has improved over time, although underrepresentation persists in specific conditions and settings [84–87]. Furthermore, large gaps still remain in adequate sex and gender representation outside of the traditional binaries of male/female and women/men, and sex-specific and gender-related factors, such as parity or external exposures or behaviors, are not commonly considered [1, 88–94], despite inclusion of these factors enhancing the resolution and quality of data [60, 95].

Across diverse fields, most studies report the sex or gender composition of the sample (although often conflating the terms), but significantly fewer disaggregate outcomes or conduct sex-/gender-based analyses. For example, an audit of neuroscience and psychiatry publications found that although 70–88% of articles published between 2019–2022 reported some consideration of sex or gender, only 5–20% treated sex as a discovery variable [72]. In cancer trials with a patient-reported outcome (PRO) endpoint, most trials (96.6%) did not report sex/gender breakdowns, only a third disaggregated data, and only 3.3% disaggregated PROs [96]. The antihypertensive drug evidence base underpinning major hypertension guidelines rarely incorporates sex/gender in study design, analysis or reporting [97]. Comparable gaps have also been reported in stroke RCTs, where only a minority report primary outcomes by sex [98].

Methodological and analytic practices contribute to these gaps. Sex differences are often misreported and inferred from one group being “significant” and the other “non-significant,” rather than testing the appropriate interaction [99]. However, when sex/gender is evaluated more rigorously it is frequently influential: in a review of 2020 psychopharmacology neuroimaging studies, only 20% adequately evaluated sex differences, but within that subset 72% reported at least one significant sex effect [100]. Treating sex/gender solely as a covariate can also obscure interpretation and hinder identification of sex- and gender-specific effects [101], and although limited statistical power is frequently cited as a rationale for omission, simulations and design guidance show that prespecified factorial models incorporating sex can be efficient and do not necessarily require doubling sample sizes [102, 103].

If organisms or individuals of one sex are underrepresented in a study, the absence of an observed sex difference should not be equated with evidence of equivalence [104], highlighting the importance of pre-specified interaction testing and sex-disaggregated analysis. Failure to meaningfully incorporate sex and gender can also slow translation into practice; for example, post-hoc analyses of an RCT of lecanemab in early Alzheimer’s disease suggest sex differences in response [105, 106], while sex differences in pharmacokinetics and pharmacodynamics contribute to differing risk of hospital admission due to adverse drug reactions in men and women [107], underscoring the value of sex- and gender-specific outcome reporting and interpretation.

## Scientific and clinical implications

Ignoring sex and gender limits scientific understanding and undermines clinical translation. Diagnostic accuracy, biomarker discovery, treatment response, and real-world outcomes are all enhanced when sex and gender are appropriately incorporated into research. Conversely, exclusion or superficial analysis perpetuates gaps in women’s health [108], results in worse outcomes in male individuals with female-predominant conditions such as breast cancer [109] osteoporosis [110], or eating disorders [111], marginalizes pregnant and lactating populations, [112] and fails to address the health needs of sexual orientation- and gender identity-diverse populations, intersex individuals and those with differences of sex development [113, 114].

Physiological differences across all domains, including blood pressure trajectories [115], cognitive performance [116], kidney function [117], and sleep and sleep-disordered breathing [118], vary across sex and gender over the life course. Yet, translation of this evidence into clinical guidelines remains limited [119], despite recent efforts to address these gaps [93, 120–127]. Without systematic integration into research design and reporting, such translational advances remain fragmented and uneven.

## OSSD position on sex and gender-responsive research with policy recommendations

While early research focused primarily on sex as a binary variable, these findings have motivated deeper investigation into underlying mechanisms, including endocrine influences, sex chromosome effects, and gender-related factors. Collectively, these findings underscore the need for health research to move beyond treating sex as a simple stratifier and toward study designs and analytic frameworks that explicitly test sex- and gender-linked pathways with the ultimate goal of more tailored clinical decision-making.

OSSD affirms that meaningful integration of sex and gender is essential to high-quality science. OSSD expects that sex and gender considerations are addressed deliberately and transparently across all stages of research, unless a strong, evidence-based justification for exclusion is provided. OSSD discourages the routine use of sex or gender solely as covariates or confounders without scientific rationale and recommends their use as discovery variables whenever feasible and appropriately powered.

OSSD recommends the following minimum expectations for sex- and gender-responsive research:

## National strategies, priority setting and funding calls

- Require sex- and gender-responsive research questions and justifications
- Include sex/gender as evaluation criteria in grant review, supported by guidance and reviewer education
- Require evidence of sex- and gender-responsive research in progress reports

## Study design and methods

- Require sex- and gender-responsive research questions and appropriate justifications
- Plan sample size and power to support sex- and gender-based analyses
- Use appropriate and validated measurement tools to incorporate sex- and gender-based factors

## Data collection and management

- Standardize reporting and coding of sex- and gender-responsive variables
- Collect relevant sex- and gender-related variables (e.g., sex chromosome complement of cells, sex of organism or participants and how this was determined, hormone status and exposure, pregnancy history, self-identified gender identity, roles and relations, sexual orientation)
- Protect participant privacy, particularly for individuals of minoritized sexual orientation and gender identity

## Analysis, reporting, and knowledge dissemination

- Prespecify sex- and gender-based analyses
- Interpret findings with transparency and within appropriate biological and social context
- Clearly acknowledge populations not included
- Disseminate findings to affected communities using gender-transformative approaches

## Roles and responsibilities across the research ecosystem

Achieving meaningful integration of sex and gender requires shared accountability across the research ecosystem. Some funders have incorporated sex and gender as evaluation criteria in grant review and provided educational materials to reviewers. However, more systematic, standardized, and sustained educational approaches, such as structured workshops and training programs, are needed to support consistent implementation and to keep practices aligned with evolving science and a changing society (e.g., shifting norms, language, and understandings of sex and gender).

OSSD recommends the following minimum expectations for building, protecting, and advancing sex- and gender-responsive research ecosystems:

## Funders and regulators

- Mandate sex- and gender-responsive research practices
- Provide clear guidance, with consistent tools and review criteria
- Define strategies for accountability beyond review (e.g., progress reports, post-marketing surveillance)

## Research institutions

- Build capacity through training of researchers, trainees, staff, and statisticians
- Integrate sex and gender concepts into curricula
- Integrate sex and gender expectations into institutional research ethics board review

## Journals and editors

- Require adherence to sex- and gender-reporting guidelines (e.g., SAGER)
- Enforce standards during peer review, including explicit evaluation criteria

## Researchers

- Engage diverse perspectives and expertise
- Commit to sex- and gender-sensitive study design and analytical methods
- Commit to continuous learning in sex- and gender-responsive science
- Commit to training students and early career researchers in sex- and gender- responsive science.

## Implementation, monitoring, and evaluation

- Use phased implementation strategies with defined milestones
- Invest in training, incentives, and infrastructure
- Track measurable indicators and publicly report progress (e.g., proportion of funded projects with robust sex/gender plans, number of resulting publications with comprehensive sex/gender data)
- Regularly revise policies based on emerging evidence [128].

## Conclusion and call to action

OSSD calls on journals, funders, institutions, regulators and investigators to move beyond minimal compliance toward rigorous, accountable implementation of sex- and gender-responsive research practices (Table 2). Without such action, critical discoveries will continue to be missed, inequities perpetuated, and scientific resources wasted. Twenty years after its founding, OSSD reaffirms that integrating sex and gender is not aspirational but essential for scientific rigor and equitable health outcomes (Fig. 1). Incorporation of sex and gender as biological and sociocultural variables is fundamental to rigorous, reproducible, and generalizable science, and represents a scientific necessity independent of shifting geographic, sociocultural, or political contexts [129]. With coordinated policy enforcement, training, and evaluation, sex and gender science can fulfill its promise to improve health outcomes for all populations.

**Table 2:** Common Pitfalls and Best Practices in Sex and Gender Research, by Study Stage

Study stage	Common pitfall	Best practice
Planning	Treating sex/gender considerations as a late-stage add-on	Integrate sex/gender from the start, including in aims, recruitment, endpoints, analysis plan, and reporting checklists
	Conflating sex (biological) and gender (social) or using terms interchangeably	Define sex and gender explicitly; measure and analyze them appropriately (and separately when possible)
	Measuring gender poorly (unclear construct, non-validated items)	Use validated gender-related measures when feasible; match the measure (identity/roles/norms) to the research question
	Using binary-only categories by default without noting limits	Use inclusive, clearly defined categories; report how sex was determined and how gender was measured; be transparent about limits
Design / Materials/ Conduct	Not reporting sex chromosomal complement of cells/tissues or using cell lines with unknown/mislabeled sex	Report cell line provenance and sex; authenticate lines; include sex as a design factor for primary cell work
	Preclinical work uses a single strain/age while claiming generalizability	Include sex across relevant ages/models; justify external validity limits
	Lack of justification for single sex/gender studies	Clear, evidence-based rationale for exclusion
	Assuming females are “more variable” as a reason to exclude from study	Use evidence-based variability assumptions; model variability rather than excluding
	Not accounting for hormonal status when it plausibly matters (e.g., cycle phase, pregnancy, menopause, hormone therapy exposure)	Measure and report relevant hormonal variables; incorporate timing/stratification/sensitivity analyses in the design
	Underpowered subgroup comparisons leading to “no difference” conclusions	Plan power for sex/gender-stratified analyses (or justify exploratory analyses); report uncertainty (confidence intervals/effect sizes), not only p-values
	Inconsistent reporting of sample size by sex/gender	Transparent reporting of sample composition
Analysis	Inclusion of males and females without analysis by sex/gender	Prespecified sex/gender-based analyses with adequate power
	Treating sex/gender as covariates	Use of sex/gender as discovery variables when appropriate
	Multiple testing across many outcomes without a plan	Prespecify primary sex/gender hypotheses; address multiplicity (e.g., adjustments or hierarchical plans)
	Pooling across sex/gender after a low-power “no interaction” test	Treat interaction tests as low-power; use estimation (effect sizes) and sensitivity analyses before pooling
	Failure to control for sex-specific confounders (e.g., body size)	Predefine confounders/mechanisms; use appropriate adjustment strategies and report model choices transparently
Reporting	Sex/gender results reported in supplementary data of publications	Integration of findings into main results and interpretation
	Reporting sex or gender differences without discussing clinical/practical relevance	Report absolute effects and decision thresholds; discuss implications for diagnosis, dosing, or risk stratification
Design / Sampling	Assuming “include both sexes” implies a simple 50/50 split regardless of sex/gender-specific incidence/prevalence, age, or reproductive stage	Plan sampling to support valid inference (including unequal recruitment when warranted) and account for age, reproductive stage/cycle, and relevant contexts
Design / Randomization	Randomization without ensuring balance within sex/gender strata	Use sex/gender-stratified randomization or factorial designs when appropriate to maintain comparability within each sex/gender
Conduct / Data collection	Collecting sex/gender as overly simplistic fields without documenting assignment/measurement and relevant clinical context	Use clear definitions and capture how sex was assigned and how gender was measured; document clinically relevant context when needed
Conduct / Data collection	Incomplete characterization of menstrual/cycling-related factors (e.g., contraceptive use, lactation) when relevant	Record menstrual timing and key modifiers (e.g., hormonal contraception, breastfeeding) when they may affect outcomes
Analysis / Interpretation	Overinterpreting statistically significant sex/gender differences in very large datasets that may be clinically trivial	Prioritize effect sizes, uncertainty, and practical/clinical relevance; interpret small effects cautiously
Analysis / Multiple comparisons	Post hoc “sex/gender-difference” fishing across many outcomes without guarding against inflated false positives	Prespecify hypotheses where possible, address multiplicity, and interpret exploratory subgroup findings conservatively
Analysis / Covariates	Adjusting for variables (e.g., body size) that may mediate sex/gender effects, obscuring pathways	Justify adjustment using causal reasoning; distinguish confounders from potential mediators and report sensitivity analyses
Reporting / Transparency	Not providing sex/gender-stratified estimates unless interaction tests are powered	Report sex/gender-stratified estimates (with uncertainty) and clearly label exploratory results to support synthesis and future studies

## Data Availability

Data sharing is not applicable to this article as no datasets were generated or analyzed.
